# Assessment of SARS-CoV-2 Infection Prevalence in Homeless Shelters — Four U.S. Cities, March 27–April 15, 2020

**DOI:** 10.15585/mmwr.mm6917e1

**Published:** 2020-05-01

**Authors:** Emily Mosites, Erin M. Parker, Kristie E. N. Clarke, Jessie M. Gaeta, Travis P. Baggett, Elizabeth Imbert, Madeline Sankaran, Ashley Scarborough, Karin Huster, Matt Hanson, Elysia Gonzales, Jody Rauch, Libby Page, Temet M. McMichael, Ryan Keating, Grace E. Marx, Tom Andrews, Kristine Schmit, Sapna Bamrah Morris, Nicole F. Dowling, Georgina Peacock, COVID-19 Homelessness Team, COVID-19 Homelessness Team, Ann Buff, Calla Jamison, Ruthanne Marcus, Carol Y. Rao, Julie L. Self, Farrell Tobolowsky, Samantha Williams, Meagan Kay, Naveena Bobba, Stephanie Cohen, Jonathan Fuchs, Trang Nguyen, Julie Stoltey

**Affiliations:** ^1^CDC COVID-19 Emergency Response; ^2^Boston Health Care for the Homeless Program; ^3^Boston Medical Center; ^4^Massachusetts General Hospital, Boston, Massachusetts; ^5^Harvard Medical School, Boston, Massachusetts; ^6^University of California San Francisco; ^7^San Francisco Department of Public Health; ^8^Public Health – Seattle & King County, Washington; ^9^Epidemic Intelligence Service, CDC; ^10^St. Joseph’s Health System, Atlanta, Georgia.; CDC COVID-19 Emergency Response; CDC COVID-19 Emergency Response; CDC COVID-19 Emergency Response; CDC COVID-19 Emergency Response; CDC COVID-19 Emergency Response; CDC COVID-19 Emergency Response; CDC COVID-19 Emergency Response; Public Health – Seattle & King County; San Francisco Department of Public Health; San Francisco Department of Public Health; San Francisco Department of Public Health; San Francisco Department of Public Health; San Francisco Department of Public Health.

In the United States, approximately 1.4 million persons access emergency shelter or transitional housing each year (*1*). These settings can pose risks for communicable disease spread. In late March and early April 2020, public health teams responded to clusters (two or more cases in the preceding 2 weeks) of coronavirus disease 2019 (COVID-19) in residents and staff members from five homeless shelters in Boston, Massachusetts (one shelter); San Francisco, California (one); and Seattle, Washington (three). The investigations were performed in coordination with academic partners, health care providers, and homeless service providers. Investigations included reverse transcription–polymerase chain reaction testing at commercial and public health laboratories for SARS-CoV-2, the virus that causes COVID-19, over approximately 1–2 weeks for residents and staff members at the five shelters. During the same period, the team in Seattle, Washington, also tested residents and staff members at 12 shelters where a single case in each had been identified. In Atlanta, Georgia, a team proactively tested residents and staff members at two shelters with no known COVID-19 cases in the preceding 2 weeks. In each city, the objective was to test all shelter residents and staff members at each assessed facility, irrespective of symptoms. Persons who tested positive were transported to hospitals or predesignated community isolation areas.

Overall, 1,192 residents and 313 staff members were tested in 19 homeless shelters (Table). When testing followed identification of a cluster, high proportions of residents and staff members had positive test results for SARS-CoV-2 in Seattle (17% of residents; 17% of staff members), Boston (36%; 30%), and San Francisco (66%; 16%). Testing in Seattle shelters where only one previous case had been identified in each shelter found a low prevalence of infection (5% of residents; 1% of staff members). Among shelters in Atlanta where no cases had been reported, a low prevalence of infection was also identified (4% of residents; 2% of staff members). Community incidence in the four cities (the average number of reported cases in the county per 100,000 persons per day during the testing period) varied, with the highest (14.4) in Boston and the lowest (5.7) in San Francisco (*2*).

**TABLE:** SARS-CoV-2 testing among residents and staff members at 19 homeless shelters in four U.S. cities with community transmission of COVID-19, March 27–April 15, 2020

City	No. of shelters assessed	Date of testing	Residents		Staff members	
			No. tested	No. (%) positive	No. tested	No. (%) positive
**Shelters reporting ≥2 cases in 2 weeks preceding testing**						
Seattle	3	Mar 30–Apr 8	179	31 (17)	35	6 (17)
Boston	1	Apr 2–3	408	147 (36)	50	15 (30)
San Francisco	1	Apr 4–15	143	95 (66)	63	10 (16)
Subtotal	5	March 30–Apr 15	730	273 (37)	148	31 (21)
**Shelters reporting 1 case in 2 weeks preceding testing**						
Seattle	12	Mar 27–Apr 15	213	10 (5)	106	1 (1)
**Shelters reporting no cases in 2 weeks preceding testing**						
Atlanta	2	Apr 8–9	249	10 (4)	59	1 (2)
**Total**	**19**	**Mar 27**–**Apr 15**	**1,192**	**293 (25)**	**313**	**33 (11)**

**Abbreviation:** COVID-19 = coronavirus disease 2019.

The findings in this report are subject to at least three limitations. First, testing represented a single time point. Second, although testing all residents and staff members at each shelter was the objective, some were not available or declined (e.g., in San Francisco 143 of an estimated 255 residents at risk were tested). Finally, symptom information for persons tested was not consistently available and thus not included, although symptom information from Boston is available elsewhere.*

Homelessness poses multiple challenges that can exacerbate and amplify the spread of COVID-19. Homeless shelters are often crowded, making social distancing difficult. Many persons experiencing homelessness are older or have underlying medical conditions (*1*,*3*), placing them at higher risk for severe COVID-19–associated illness (*4*).

To protect homeless shelter residents and staff members, CDC recommends that homeless service providers implement recommended infection control practices, apply social distancing measures including ensuring residents’ heads are at least 6 feet (2 meters) apart while sleeping, and promote use of cloth face coverings among all residents.^†^ These measures become especially important once ongoing COVID-19 transmission is identified within communities where shelters are located. Given the high proportion of positive tests in the shelters with identified clusters and evidence for presymptomatic and asymptomatic transmission of SARS-CoV-2 (*5*), testing of all residents and staff members regardless of symptoms at shelters where clusters have been detected should be considered. If testing is easily accessible, regular testing in shelters before identifying clusters should also be considered. Testing all persons can facilitate isolation of those who are infected to minimize ongoing transmission in these settings.

## Footnotes

* https://www.medrxiv.org/content/10.1101/2020.04.12.20059618v1.

† https://www.cdc.gov/coronavirus/2019-ncov/community/homeless-shelters/plan-prepare-respond.html.
